# Private health insurance in the United States and Sweden: A comparative review

**DOI:** 10.1002/hsr2.1979

**Published:** 2024-03-14

**Authors:** Udit Dave, Emma G. Lewis, Jenilkumar H. Patel, Nikhil Godbole

**Affiliations:** ^1^ Tulane University School of Medicine New Orleans Louisiana USA

**Keywords:** health insurance, Medicare, Sweden, United States, universal health care

## Abstract

**Background and Aims:**

The United States of America and Sweden both contain a public and private component to their healthcare systems. While both countries spend a similar amount per capita on public healthcare expenditures, the United States spends significantly more in the private healthcare sector. Sweden has a social democratic model of health care, and given its identity as a welfare state, private health insurance providers have a small and nuanced role.

**Methods:**

This paper was completed after searches were queried for “Sweden,” “United States,” and variants of the words “insurance,” “public,” “private,” “Medicare,” “Medicaid,” “public,” and “costs.” A preliminary search in May 2022, yielded 78 articles, of which 45 were ultimately considered relevant for this review. Inclusion criteria consisted of English language articles, topic relevance, and verification of MEDLINE‐indexed journals. These searches were performed in PubMed, Google Scholar, Embase, and Cochrane. Summary findings of these searches are compiled in this review.

**Results:**

Sweden guarantees low‐cost appropriate care to all citizens with equitable access; however, drawbacks of its system include high financial burden, lack of primary care infrastructure, as well as geographical and socioeconomic inequities. On the other hand, the United States' healthcare system is built around the private sector with public health insurance reserved only for the most vulnerable patient populations.

**Conclusion:**

Our goal is to provide an overview, compare the role of private health insurance in both countries, and highlight policies that have had beneficial effects in each nation. Possible solutions to the drawbacks of each nation's health insurance policies could be addressed by additional support to Sweden's vulnerable population by developing a program similar to the US' Medicare Advantage program. Conversely, the United States may benefit from increasing access to public health insurance, especially in instances where families face unemployment.

## 1 INTRODUCTION

Sweden has been referenced globally as a leading example of socialized health care, as its health care system is generally associated with high levels of public spending and subsequent positive health outcomes. Meanwhile, the United States' health care system is generally criticized perceived as expensive and inefficient at delivering desired health outcomes. In recent years, Sweden has shifted towards a greater reliance on private health care and insurance due to the immense financial burdens associated with government‐funded health care.^1^ In contrast, the United States' healthcare system is heavily reliant on the private sector, with recent strides toward public insurance expansion to increase the number of Americans insured.^2^ In this study, Sweden and the United States are chosen as model systems in part due to their nearly identical per capita healthcare expenditures. This allows for effective comparison of financial allocations, government and private programs, and health care systems utilization and outcomes. Furthermore, the two nations are tokenized as nearly opposing models of healthcare delivery, with Sweden's socialist approach and the United States' privatized model. This article seeks to establish key differences that may help explain the vast differences in healthcare access and life expectancy between the two nations. In this paper, we provide an overview of both the Swedish and American healthcare systems, compare the role of private health insurance in both countries, and highlight policies that have had beneficial effects in each nation.

## 2 METHODS

A comprehensive search of PubMed, Embase, and Cochrane Library databases was performed. This paper was completed after searches were queried for “Sweden,” “United States,” and variants of the words “insurance,” “public,” “private,” “Medicare,” “Medicaid,” “public,” and “costs.” A comprehensive preliminary search of PubMed, Embase, Cochrane Library, and Google Scholar for articles published from 1998 to May 2022 was performed., This search query yielded 78 articles, of which 45 were ultimately considered relevant for this review. Inclusion criteria consisted of English language articles, topic relevance, and verification of MEDLINE indexed journals. These searches were performed in PubMed, Google Scholar, Embase, and Cochrane. Summary findings of these searches are compiled in this review. Data quality assessment was performed with the JBI Critical Appraisal Tool.^3^, ^4^, ^5^ Full texts were reviewed based on inclusion and exclusion criteria to ascertain validity and reasonably low levels of potential bias. The aforementioned assessment tool was then used to grade study quality.

## 3 COMPARISON OF HEALTHCARE SYSTEMS

### 3.1 Overview of the Swedish healthcare system

Sweden established a social democratic model of health care beginning in the 1970s and 1980s with the primary goal of providing equitable medical care to all individuals.^6^ Beginning in the 1990s, private insurance emerged as a major player in health care delivery. Presently, approximately 10% of working‐age Swedes are subscribed to a private health insurance plan.^6^ On the other hand, public spending accounts for 84% of total healthcare spending in Sweden.^7^ Total health care spending per capita in Sweden is nearly 5500 USD per person per year.^8^

Private health insurance covers forms of care that are not covered by public plans. This includes specialty programs such as chiropractic care, dietician access, rehabilitation, and at‐home postsurgical care.^6^ Most private health insurance providers also offer health care planning and counseling services, public usage fee coverage, and allowances for drugs, vaccinations, dental care, and overseas care.^6^ Sweden also has a program of Voluntary Health Insurance (VHI) that is predominantly afforded by private sector employees and businesspeople.^9^ VHI is primarily used in a complementary setting to cover out‐of‐pocket payments associated with public health care use. It is also used less frequently in a duplicative setting to expedite access to normally free health care that may have longer than desired wait times.^10^ VHI premiums are based on health risks, meaning that individuals who would most likely desire health insurance in addition to their public coverage would be least likely to be accepted by private VHI providers.^9^ However, VHI popularity has grown over the past two decades in Sweden, especially among privileged individuals, as it provides coverage for healthcare services that are not traditionally covered by standard Swedish public health insurance.^9^ However, only 10% of Swedes between the ages of 16 and 64 own a VHI policy.^11^ Despite this potential for inequity posed by the offering of VHI policies, a previous study has shown that VHI policyholders are less likely to utilize public health care than those without a policy, reducing the burden placed on the public Swedish health care system.^12^

Sweden boasts a life expectancy of 82 years and has a low amenable mortality rate, indicating its hospital systems are effective at preventing deaths from illnesses that are deemed treatable by modern healthcare standards.^7^ One of the major causes of inefficiency facing the Swedish healthcare system is long wait times, as emphasis is placed on treating the sickest patients first.^7^, ^13^ Additionally, Sweden does not have a robust primary care system, and improvements are required in monitoring results.^14^ Furthermore, an issue at the forefront of Swedish politics is the Stop Law, which aims to prevent fast‐track lanes to healthcare access that are provided to individuals who have private coverage.^6^ Previous legislation that aimed to end fast‐tracking to avoid long wait times was inadequate, and a generally unanimous viewpoint shared by Swedish healthcare personnel is that healthcare access should be fully equal to all.^6^

### 3.2 Overview of the American healthcare system

The United States features a blend of public and private health insurance. Public programs include Medicare, which provides health insurance for elderly individuals, and Medicaid, which provides insurance for financially disadvantaged individuals. A major issue burdening the American healthcare system is a large number of underinsured and uninsured individuals. Many Americans do not qualify for public health insurance, and while employers are a major provider of private health insurance plans, these plans often do not protect against large, catastrophic health costs.^15^ To combat inadequacies in health care coverage, the Affordable Care Act was signed into law in 2010. In 5 years after the ratification of this act, the American uninsurance rate dropped from 16.0% to 9.1%.^2^

The United States has very high levels of healthcare spending. In fact, total (public and private) per capita health spending in the United States is over 10,200 USD.^8^ These high expenditures however do not necessarily result in significant improvement in mortality. This is exemplified in the nation's life expectancy of 78.6 years, which is 2 years lower than the average of countries belonging to the Organization for Economic Co‐operation and Development.^8^ A contributing factor may be the United States' low expenditures on social services, which can be beneficial in helping patients navigate the country's complex healthcare system.^16^ Furthermore, racial, and ethnic disparities in mortality rates exist in the United States, with significantly lower life expectancies for Black Americans (75.3 years).^8^ A current consideration in American health care is to reduce costs while increasing the number of insured individuals by improving access regardless of socioeconomic status. Table 1 highlights key differences between the Swedish and American healthcare systems.

**Table 1:** Summary of life expectancy and health care expenditure in Sweden and the United States.

	Sweden	United States
Life expectancy	82.0 years	78.6 years
Annual public health care expenditure (per capita)	4600 USD	5000 USD
Annual private healthcare expenditure (per capita)	100 USD	4100 USD
Annual out‐of‐pocket healthcare expenditure (per capita)	800 USD	1100 USD
Annual total healthcare expenditure (per capita)	5500 USD	10,200 USD

*Note*: Per capita costs are estimated to the nearest 100 USD.

## 4 THE ROLE OF PRIVATE HEALTH INSURANCE IN SWEDEN AND THE UNITED STATES

The United States and Sweden have very similar public healthcare spending amounts. The United States spends approximately 4993 USD annually per capita on public health care while Sweden spends about 4569 USD.^8^ However, compared to Sweden, the United States has a much greater emphasis on private health insurance and care. Private spending on health care in Sweden is roughly 71 USD per capita yearly with per capita out‐of‐pocket medical costs of 807 USD. This differs starkly from the United States' yearly estimates of 4092 USD per capita for private spending and 1122 USD per capita coming out‐of‐pocket.^8^

Under the guidance of the Affordable Care Act, private insurers in the United States have placed attention on preventative care, with extensive cancer screening programs saving millions of American lives annually.^2^ The robust healthcare expenditures in the United States are likely driven by the utilization of expensive diagnostic technology for preventative screening such as magnetic resonance imaging (MRIs). These prices are further exacerbated by the general cost of the numerous healthcare services that come with preventative practice, which in many circumstances are driven by high administrative costs.^16^ Interestingly, compared to countries with lower private and out‐of‐pocket healthcare costs, the United States does not have an increased frequency in healthcare utilization or hospital admission, which suggests that high costs are not driven by overutilization of resources.^16^

Compared to the United States, in which public health care coverage is reserved for vulnerable populations and private insurance covers most individuals, Swedish private health care firms compete directly with public facilities to provide care to patients.^1^ Despite Sweden's national focus on guaranteed universal health care access, there is country‐wide interest in potentially supplementing the public system with access to privatized hospital care and private health insurance—a shift that is supported by both physician and nursing unions.^17^ Recent private insurance trends also include coverage of dental procedures, drug expenses, co‐payments, psychiatric treatment, and costs associated with loss of income due to illness.^18^ However, these shifts in privatization have been accompanied by a widening of inequities by sociodemographic factors in Sweden with less ability to control the quality‐of‐care patients receive.^14^ Citizens are less satisfied with the high tax rate they pay for universal health care when private options exist, and there is the potential for tax break functionalities to be abused by firms and individuals.^13^ Additionally, the present way private health insurance is utilized in Sweden does not provide support to the public system, but rather detracts from it via competition. Healthcare providers who accept private insurance often treat patient groups under agreements that differ from their contracts with local governments and private firms to provide care to local populations.^13^

## 5 POLICY CONSIDERATIONS

The United States and Sweden exhibit different blends of public and private healthcare systems. Each country faces budgeting issues and difficulties with providing quality care with equitable access at an appropriate price point. However, both countries' variable methods of insurance offer beneficial policy considerations regarding the role of private health insurance.

A potential role of private insurance is to increase health service provision to individuals in the deepest need.^14^ As the role of private health insurance has increased in Sweden in recent years, the country has faced the ethical issue of individuals with private insurance attempting to “skip the line” to possibly receive care for symptoms or conditions that may not require immediate evaluation. Alternatively, private health insurance could instead provide additional support to Swedes who are geographically or economically disadvantaged and may face barriers in access to care. Private insurance has the potential to reduce the cost burden of providing equitable care to the aforementioned at‐risk individuals. Further, the public Swedish plan could subsidize VHI policies for its socio‐geographically vulnerable populations to improve access. Additionally, public insurance coverage could be expanded for those at‐risk, covering services that are not typically fully insured in the general population including dental procedures and psychiatric care. In the United States, the passing of the Affordable Care Act has resulted in the rise of Medicare Advantage plans: private health insurance plans pre‐approved by Medicare to contribute additional coverage that supplements the public care provided by Medicare.^19^ Medicare Advantage offers a number of benefits such as vision, dental, hearing, and fitness coverage—all of which are programs not presently offered by Medicare alone.^19^ From a fiscal standpoint, Medicare Advantage generates revenues 30% greater than spending for private health insurance providers while spending between 9% and 30% less per patient compared to traditional Medicare.^20^ The Swedish healthcare system may benefit from implementing a similar program given its current system's weakness in primary care infrastructure and the Swedish reverence of equity in healthcare access.

The most common way Americans obtain private health insurance policies is through their employer, a system somewhat akin to the Swedish private sector health benefits provided by VHI programs. Previous studies have shown that individuals with access to VHI plans are socioeconomically privileged and that this program generally provides benefits for healthy and wealthy individuals.^9^ Tying health insurance coverage to employment has the potential to yield high inequities in access, especially in a global economy with frictional unemployment. In Sweden, the provision of universal health coverage may help mitigate these inequities by allowing all Swedes basic health coverage. Interestingly, one study found that those who own VHI policies and those who do not own such policies share an equitable willingness to pay taxes that finance the public healthcare system.^12^ Conversely, the United States does not present a robust public health insurance program. Many Americans are highly satisfied with the health insurance they receive as part of their compensation from their employer, but the United States should consider expanding eligibilities for public health insurance options to prevent catastrophic health outcomes that can be associated with an individual losing their job.^15^

The amount of time spent during different stages of a medical appointment has been increasing in several countries, and time constraints associated with seeking health care serve as a major hurdle for obtaining access to care.^21^ The utility of comparing waiting times between countries that use different models of health care can be limited as the calculation and definition of wait times may differ considerably from nation to nation. Sweden uses waiting‐time prioritization for rationing health care services in which patients requiring more urgent procedures receive quicker access to care, subsequently leading to increased wait times for individuals presenting with nonurgent concerns.^21^ Horizontal inequality within various Nordic healthcare systems has not been extensively researched. A Danish study established an association between lower socioeconomic status (SES) and increased waiting times for referral to a specialist provider.^22^ Conversely, a study based in Norway found no association of horizontal inequality in the context of SES and wait times.^23^ Multiple studies have looked at the relationship between SES and wait times, but they have all varied substantially in their methodology and objectives, making it challenging to draw reasonable conclusions.^24^, ^25^, ^26^, ^27^, ^28^ In Sweden, when it comes to receiving primary care, Vårdgaranti—National Guaranteed Access to Healthcare—was established on November 1, 2005. This protocol lays the standard for waiting times as it pertains to planned visits for a primary care physician (PCP) or a specialist.^29^, ^30^ This established standard of care aims to keep the waiting times for primary care visits under 7 days and for a specialist under 90 days, but it does not apply to emergency care.^29^, ^30^ In United States, studies in several states found that patients who are privately insured generally have shorter wait times for a new primary care provider (PCP) compared to publicly insured patients.^31^, ^32^ The difference in wait times between the two groups was mainly attributed to the dissimilarity in payment rates between payers, something which is inherently not advantageous to the private insurers.^32^ One of the major drawbacks of using wait times to compare private versus public insurance when it comes to patients seeking care is that it does not consider uninsured patients. There are approximately 27 million uninsured people in the United States, and these individuals are more likely to delay seeking care or required medical attention due to associated costs.^33^ A key policy consideration that the United States could incorporate into its own system would be using public funding to model Sweden's healthcare system with a specific aim towards providing equitable medical care to uninsured individuals.

Analyzing the effectiveness of healthcare systems across different nations can be complex due to differences in healthcare delivery, funding mechanisms, and the health requirements of diverse populations. Nevertheless, numerous studies have offered valuable insights into comparing the healthcare systems of Sweden and the United States. A study comparing older populations in six countries, including Sweden and the United States, revealed that healthcare systems with a state‐based approach, like Sweden's, generally leads to superior population health outcomes compared to private‐based systems like the United States. The study also identified a more pronounced positive connection between wealth and health in the United States, indicating that wealthier individuals may experience better health outcomes.^34^ A cross‐national study by Starfield compared effectiveness of primary care and health in 10 western industrialized nations based on three key indicators: 12 health indicators, extent of primary health service, and population satisfaction in relation to the overall cost of the system. Some key health indicators included infant mortality, life expectancy, and age‐adjusted death rates. This study found that the United States had low ratings on measures of primary care, health indicators, and the satisfaction‐expense ratio. In contrast, Sweden had generally high ratings for all three measures.^35^

Healthcare systems can also be compared based on administrative efficiency, including the costs associated with billing, patient enrollment, marketing, and other business aspects of healthcare. Sweden typically has lower administrative overhead and simpler billing due to its centralized publicly funded system. In contrast, the United States healthcare system faces higher administrative complexity with multiple payers, billing procedures, deductibles, and paperwork. According to a study by Papanicolas et al., the United States spends 8% of its GDP on healthcare administration costs, while Sweden only spends 2%. This highlights a significant difference in administrative expenditure between the two countries.^36^ The United States spends two to five times more on healthcare administrative costs compared to other developed countries, resulting in higher health insurance premiums for the public.^37^ In the United States, physicians experience a greater administrative burden compared to those in Sweden, but high administrative challenges are present in all insurance‐based systems. A survey showed that 54% of US physicians find time spent on insurance‐related administrative issues to be a major problem. Additionally, 33% cited reporting clinical or quality data as a major problem, and 16% reported spending a significant amount of time on paperwork or disputes related to medical bills.^36^ According to a study by Anskär et al., Swedish PCPs spent 30.9%–37.2% of their work time on administrative tasks categorized as “other work tasks.” These tasks included activities like meetings, continuing education, email management, handling equipment and facilities, addressing computer issues, waiting time, and brief refreshments break.^38^ Physicians allocate varying amounts of time to administrative tasks related to both direct and indirect patient care in the United States and Sweden. In the United States, the focus is on insurance and claims, while in Sweden, it involves psychosocial work and quantitative demands. This significant time spent on administrative duties influences how physicians perceive their capacity to deliver quality care, their career satisfaction, the risk of burnout, and the likelihood of remaining in clinical practice. The diversity in administrative burden is evident globally, with different work areas contributing to the overall workload. The substantial time devoted to administrative tasks plays a crucial role in driving variations in overall healthcare spending across countries. In Sweden, enhancing administrative efficiency includes hiring more personnel with expertise in healthcare systems, particularly in private insurance, as healthcare privatization increases, will help to reduce physicians' time spent on administrative tasks.^38^ In the United States, addressing administrative challenges could be improved by (A) simplifying prior authorization by obtaining approval from health insurers before services or prescriptions, reducing requirements, and cutting associated documentation to lower costs. (B) Standardizing billing and claims processing through a centralized clearinghouse, allowing providers to submit all claims to a single entity, and establishing uniform costs for routine procedures to enhance efficiency. (C) Ensuring proper medical coding with qualified coders to prevent wasted time and resources, correcting inaccuracies, and minimizing claim denials in provider documentation for an effective revenue cycle.^39^

Pharmaceutical and medical device companies play a crucial role in the healthcare sector as they are responsible for drug development, innovation in devices used for medical procedures, investing in research which helps in disease prevention and treatment. Biomedical research funding in the United States increased from $37.1 billion in 1994 to $94.3 billion in 2003.^40^ Recent figures show that the median investment for developing a new drug is $985.3 million, with an average investment of $1335.9 million in the United States.^41^ In Sweden, detailed information on the total investment by pharmaceutical companies in medical research is not easily accessible. However, a study on funding to patient organizations revealed that 46 pharmaceutical companies contributed €6.5 million (equivalent to 7 million USD) to 77 patient organizations from 2014 to 2018. While this offers insight, it does not capture the complete scope of research and development investment in Sweden.^42^ Per capita, the United States invests approximately $1443, while Sweden's per capita spending is $566, indicating a significant difference in pharmaceutical company investment between the two countries.^36^ In pharmaceutical innovation, the United States contributes 57% of new chemical entities globally, while there are no available estimates for Sweden. The United States also has a high generic penetration rate at 84% of the total pharmaceutical market. Despite this, the spending on generic products as a percentage of total pharmaceutical spending in the United States is comparable to other developed countries, indicating that brand‐name pharmaceuticals largely drive the overall high spending, as per a study by Papanicolas et al.^36^ A key concern for US health policymakers is the impact of the pharmaceutical market on healthcare spending. The United States invests nearly twice as much as other developed countries in pharmaceutical innovation, primarily due to the higher prices of brand‐name drugs, rather than increased utilization.^36^, ^43^, ^44^, ^45^ The significant spending variations between the United States and Sweden, and other developed nations, may be attributed to the profit‐driven motives of pharmaceutical companies, with utilization rates remaining relatively stable.^36^, ^46^, ^47^ Policy recommendations for both countries should emphasize promoting the endorsement of generic drugs by physicians and pharmacists, given their bioequivalence to brand‐name products. Additionally, facilitating the streamlined entry of generic drugs to the market after meeting necessary testing requirements could be a significant legislative improvement.

## 6 CONCLUSION

In conclusion, the role of private health care varies greatly in the United States and Sweden. In ongoing debates regarding resolution of issues pertaining to costs, access, and equity in the healthcare arenas in both countries, the utilization of private healthcare is often at the center. Both countries can glean important policy considerations from an examination of the other's healthcare systems.

## AUTHOR CONTRIBUTIONS

All authors have read and approved the final version of the manuscript. All authors, including the corresponding author Nikhil Godbole, had full access to all of the data in this study and take complete responsibility for the integrity of the data and the accuracy of the data analysis.

## CONFLICT OF INTEREST STATEMENT

The authors declare no conflict of interest.

## TRANSPARENCY STATEMENT

The lead author Nikhil Godbole affirms that this manuscript is an honest, accurate, and transparent account of the study being reported; that no important aspects of the study have been omitted; and that any discrepancies from the study as planned (and, if relevant, registered) have been explained.

## Supporting information

Supporting information.

## Data Availability

The authors have nothing to report.
