# Supplementary material for: Private health insurance in the United States and Sweden: A comparative review
Source: Health Sci Rep. 2024 Mar 14;7(3):e1979. doi: 10.1002/hsr2.1979 (PMC10940498; doi:10.1002/hsr2.1979)
Supplement: Supplementary file 1 — Supporting information. [file HSR2-7-e1979-s001.docx]

**JBI Critical Appraisal Tool**

**Policy studies.^43^**

| **Policy Studies** | **Kullbery 2019** | **Alexandersen 2016** | **Kullberg 2021** | **Medicare 2021** | **Vårdgaranti 2022** | **Väntetider 2022** |
| --- | --- | --- | --- | --- | --- | --- |
|  |  |  |  |  |  |  |
| **Are the developers of the policy/ consensus guideline (and any allegiences/affiliations) clearly identified?** | **1** | **1** | **1** | **1** | **1** | **1** |
| **Do the developers of the policy/ consensus guideline have standing in the field of expertise?** | **0** | **0** | **1** | **1** | **1** | **1** |
| **Are appropriate stakeholders involved in developing the policy/guideline and do the conclusions drawn represent the views of their intended users?** | **1** | **1** | **1** | **1** | **1** | **1** |
| **Are biases due to competing interests acknowledged and responded to?** | **1** | **1** | **1** | **1** | **1** | **1** |
| **Are the processes of gathering and summarizing the evidence described?** | **1** | **1** | **1** | **1** | **1** | **1** |
| **Is any incongruence with the extant literature/evidence logically defended?** | **1** | **1** | **0** | **1** | **1** | **1** |
| **Are the methods used to develop recommendations described?** | **1** | **0** | **0** | **1** | **1** | **1** |
|  |  |  |  |  |  |  |
| **Total** | **6** | **5** | **5** | **7** | **7** | **7** |

**Expert Opinion.^43^**

| **Expert opinion Study** | **Mason 2008** | **Obama 2016** | **Lapidus 2017** | **Rice 2016** | **Lapidus 2020** | **Dahlgren 2014** | **Anell 2005** | **Tynkkynen 2018** | **Curto 2019** | **Pettinger 1999** | **Papanicolas 2018** |
| --- | --- | --- | --- | --- | --- | --- | --- | --- | --- | --- | --- |
|  |  |  |  |  |  |  |  |  |  |  |  |
| **Is the source of the opinion clearly identified?** | **1** | **1** | **1** | **1** | **1** | **1** | **1** | **1** | **1** | **1** | **1** |
| **Does the source of opinion have standing in the field of expertise?** | **1** | **1** | **1** | **1** | **1** | **0** | **1** | **1** | **0** | **0** | **1** |
| **Are the interests of the relevant population the central focus of the opinion?** | **1** | **1** | **1** | **1** | **1** | **1** | **0** | **1** | **1** | **1** | **1** |
| **Does the opinion demonstrate a logically defended argument to support the conclusions drawn?** | **1** | **1** | **1** | **1** | **1** | **1** | **1** | **1** | **1** | **1** | **1** |
| **Is there reference to the extant literature?** | **1** | **1** | **1** | **1** | **1** | **1** | **1** | **1** | **1** | **1** | **1** |
| **Is any incongruence with the literature/sources logically defended?** | **1** | **1** | **1** | **0** | **1** | **0** | **1** | **0** | **1** | **1** | **1** |
|  |  |  |  |  |  |  |  |  |  |  |  |
| **Total** | **6** | **6** | **6** | **5** | **6** | **4** | **5** | **5** | **5** | **5** | **6** |

| **Expert opinion Study** | **Oroma 2016** | **Anskär 2018** | **Ryan 2022** | **Moses 2005** | **Wouters 2020** | **Mulinari 2020** | **Wouters 2017** | **Sarnak 2017** | **O’Neill** | **Anderson 2003** | **Dieleman 2017** |
| --- | --- | --- | --- | --- | --- | --- | --- | --- | --- | --- | --- |
|  |  |  |  |  |  |  |  |  |  |  |  |
| **Is the source of the opinion clearly identified?** | **1** | **1** | **1** | **1** | **1** | **1** | **1** | **1** | **1** | **1** | **1** |
| **Does the source of opinion have standing in the field of expertise?** | **0** | **0** | **0** | **0** | **1** | **0** | **1** | **0** | **0** | **0** | **1** |
| **Are the interests of the relevant population the central focus of the opinion?** | **1** | **1** | **1** | **1** | **1** | **1** | **1** | **1** | **1** | **1** | **1** |
| **Does the opinion demonstrate a logically defended argument to support the conclusions drawn?** | **1** | **1** | **1** | **1** | **1** | **1** | **1** | **1** | **1** | **1** | **1** |
| **Is there reference to the extant literature?** | **1** | **1** | **0** | **1** | **1** | **1** | **1** | **1** | **1** | **1** | **1** |
| **Is any incongruence with the literature/sources logically defended?** | **1** | **1** | **1** | **1** | **1** | **1** | **1** | **1** | **1** | **1** | **1** |
|  |  |  |  |  |  |  |  |  |  |  |  |
| **Total** | **5** | **5** | **4** | **5** | **6** | **5** | **6** | **5** | **5** | **5** | **6** |

**Narrative Study.^43^**

| **Narrative Study** | **McIntyre 2020** |
| --- | --- |
|  |  |
| **Is the generator of the narrative a credible or appropriate source?** | **1** |
| **Is the relationship between the text and its context explained? (where, when, who with, how)** | **1** |
| **Does the narrative present the events using a logical sequence so the reader or listener can understand how it unfolds?** | **1** |
| **Do you, as reader or listener of the narrative, arrive at similar conclusions to those drawn by the narrator?** | **1** |
| **Do the conclusions flow from the narrative account?** | **1** |
| **Do you consider this account to be a narrative?** | **1** |
|  |  |
| **Total** | **6** |

**Economic Evaluations.^44^**

| **Economic Evaluations Study** | **OECD 2017** | **Tikkanen 2019** | **Claxton 2015** | **Squire 2015** | **Berchick 2019** |
| --- | --- | --- | --- | --- | --- |
|  |  |  |  |  |  |
| **Is there a well-defined question?** | **1** | **1** | **1** | **1** | **1** |
| **Is there comprehensive description of alternatives?** | **0** | **1** | **0** | **0** | **1** |
| **Are all important and relevant costs and outcomes for each alternative identified?** | **0** | **1** | **1** | **1** | **1** |
| **Has clinical effectiveness been established?** | **1** | **0** | **1** | **1** | **1** |
| **Are costs and outcomes measured accurately?** | **1** | **1** | **1** | **1** | **1** |
| **Are costs and outcomes valued credibly?** | **1** | **0** | **1** | **1** | **1** |
| **Are costs and outcomes adjusted for differential timing?** | **1** | **0** | **1** | **1** | **1** |
| **Is there an incremental analysis of costs and consequences?** | **1** | **1** | **1** | **1** | **1** |
| **Were sensitivity analyses conducted to investigate uncertainty in estimates of cost or consequences?** | **1** | **1** | **0** | **1** | **1** |
| **Do study results include all issues of concern to users?** | **1** | **1** | **1** | **1** | **1** |
| **Are the results generalizable to the setting of interest in the review?** | **1** | **1** | **1** | **1** | **1** |
|  |  |  |  |  |  |
| **Total** | **9** | **8** | **9** | **10** | **11** |

**Cross-sectional Studies.^45^**

| **Cross-Sectional Study** | **Sorensen 2009** | **Yong 2004** |
| --- | --- | --- |
|  |  |  |
| **Were the criteria for inclusion in the sample clearly defined?** | **1** | **1** |
| **Were the study subjects and the setting described in detail?** | **1** | **1** |
| **Was the exposure measured in a valid and reliable way?** | **1** | **1** |
| **Were objective, standard criteria used for measurement of the condition?** | **1** | **1** |
| **Were confounding factors identified?** | **1** | **1** |
| **Were strategies to deal with confounding factors stated?** | **1** | **1** |
| **Were the outcomes measured in a valid and reliable way?** | **1** | **1** |
| **Was appropriate statistical analysis used?** | **1** | **1** |
|  |  |  |
| **Total** | **8** | **8** |

**Cohort Studies.^45^**

| **Cohort Study** | **Arnesen 2002** | **Pell 2000** | **Hacker 2004** | **Shortt 2003** | **Tipirneni 2015** | **Saloner 2019** |
| --- | --- | --- | --- | --- | --- | --- |
|  |  |  |  |  |  |  |
| **Were the two groups similar and recruited from the same population?** | **1** | **1** | **1** | **1** | **1** | **1** |
| **Were the exposures measured similarly to assign people to both exposed and unexposed groups?** | **1** | **1** | **1** | **1** | **0** | **1** |
| **Was the exposure measured in a valid and reliable way?** | **1** | **1** | **1** | **1** | **1** | **1** |
| **Were confounding factors identified?** | **1** | **1** | **1** | **1** | **0** | **1** |
| **Were strategies to deal with confounding factors stated?** | **1** | **1** | **1** | **1** | **1** | **1** |
| **Were the groups/participants free of the outcome at the start of the study (or at the moment of exposure)?** | **1** | **1** | **1** | **1** | **1** | **1** |
| **Were the outcomes measured in a valid and reliable way?** | **1** | **1** | **1** | **1** | **1** | **1** |
| **Was the follow up time reported and sufficient to be long enough for outcomes to occur?** | **1** | **1** | **1** | **1** | **1** | **1** |
| **Was follow up complete, and if not, were the reasons to loss to follow up described and explored?** | **1** | **1** | **1** | **1** | **1** | **1** |
| **Were strategies to address incomplete follow up utilized?** | **1** | **1** | **1** | **1** | **1** | **1** |
| **Was appropriate statistical analysis used?** | **1** | **1** | **1** | **1** | **1** | **1** |
|  |  |  |  |  |  |  |
| **Total** | **11** | **11** | **11** | **11** | **9** | **11** |
